# Transcription Factors in Heart: Promising Therapeutic Targets in Cardiac Hypertrophy

**DOI:** 10.2174/157340311799960618

**Published:** 2011-11

**Authors:** Shrey Kohli, Suchit Ahuja, Vibha Rani

**Affiliations:** Department of Biotechnology, Jaypee Institute of Information Technology, NOIDA 201307, India

**Keywords:** Cardiac hypertrophy, pro-hypertrophic, anti-hypertrophic, cardiac transcription factors, therapeutic targets, heart failure.

## Abstract

Regulation of gene expression is central to cell growth, differentiation and diseases. Context specific and signal dependent regulation of gene expression is achieved to a large part by transcription factors. Cardiac transcription factors regulate heart development and are also involved in stress regulation of the adult heart, which may lead to cardiac hypertrophy. Hypertrophy of cardiac myocytes is an outcome of the imbalance between prohypertrophic factors and anti-hypertrophic factors. This is initially a compensatory mechanism but sustained hypertrophy may lead to heart failure. The growing knowledge of transcriptional control mechanisms is helpful in the development of novel therapies. This review summarizes the role of cardiac transcription factors in cardiac hypertrophy, emphasizing their potential as attractive therapeutic targets to prevent the onset of heart failure and sudden death as they can be converging targets for current therapy.

## INTRODUCTION

Transcription factors are sequence-specific DNA binding proteins that control the process of transcription [1, 2]. They contain one or more DNA-binding domains which help them to attach to specific sequences of DNA adjacent to genes [3, 4]. Besides, other proteins such as coactivators are also involved in the regulation of transcription by binding to the transcription factors. [5].

Transcription factors (TFs) are classified into two broad categories viz., General Transcription Factors (GTFs) and Gene Specific Transcription factors (GSTFs). GTFs are involved in formation of the pre-initiation complex that binds to the promoter region of DNA and regulates the basal transcriptional regulation [6]. On the other hand, GSTFs bind to sequences specific to certain genes and thus contribute to differential gene expression [7, 8]. An understanding of altered gene expression in diseased cells lays the foundation for novel therapeutic strategies involving manipulation of gene expression. Targeting transcription factors represents one such innovation that can allow researchers to manipulate gene activation or suppression in a specific fashion, despite which there exist challenges to target them therapeutically and most approaches alter their activity indirectly. Research at the chemical biology interface has paved way for the development of new methodologies for targeting transcription factors including blocking transcription factor dimerisation, targeting specific DNA sequences and DNA decoys [9].

**Fig. (1):**
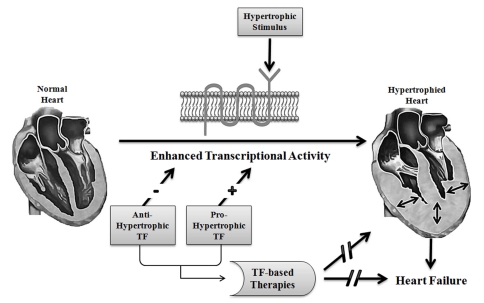
**General mechanism of cardiac hypertrophy and its effect on heart.** Following a hypertrophic stimulus, there is an enhanced transcriptional activity of disease causing marker genes, and the consequential physiological changes including augmented protein synthesis, increased myocyte size, sarcomeric reorganization, reduction in the cardiac chamber size, and ventricular wall thickening. Both, Pro- Hypertrophic TFs and Anti-Hypertrophic TFs can be used as therapeutic targets to affect a block in the induction of hypertrophic signalling cascades, and hence preventing the manifestation of the disease.

**Fig. (2).**
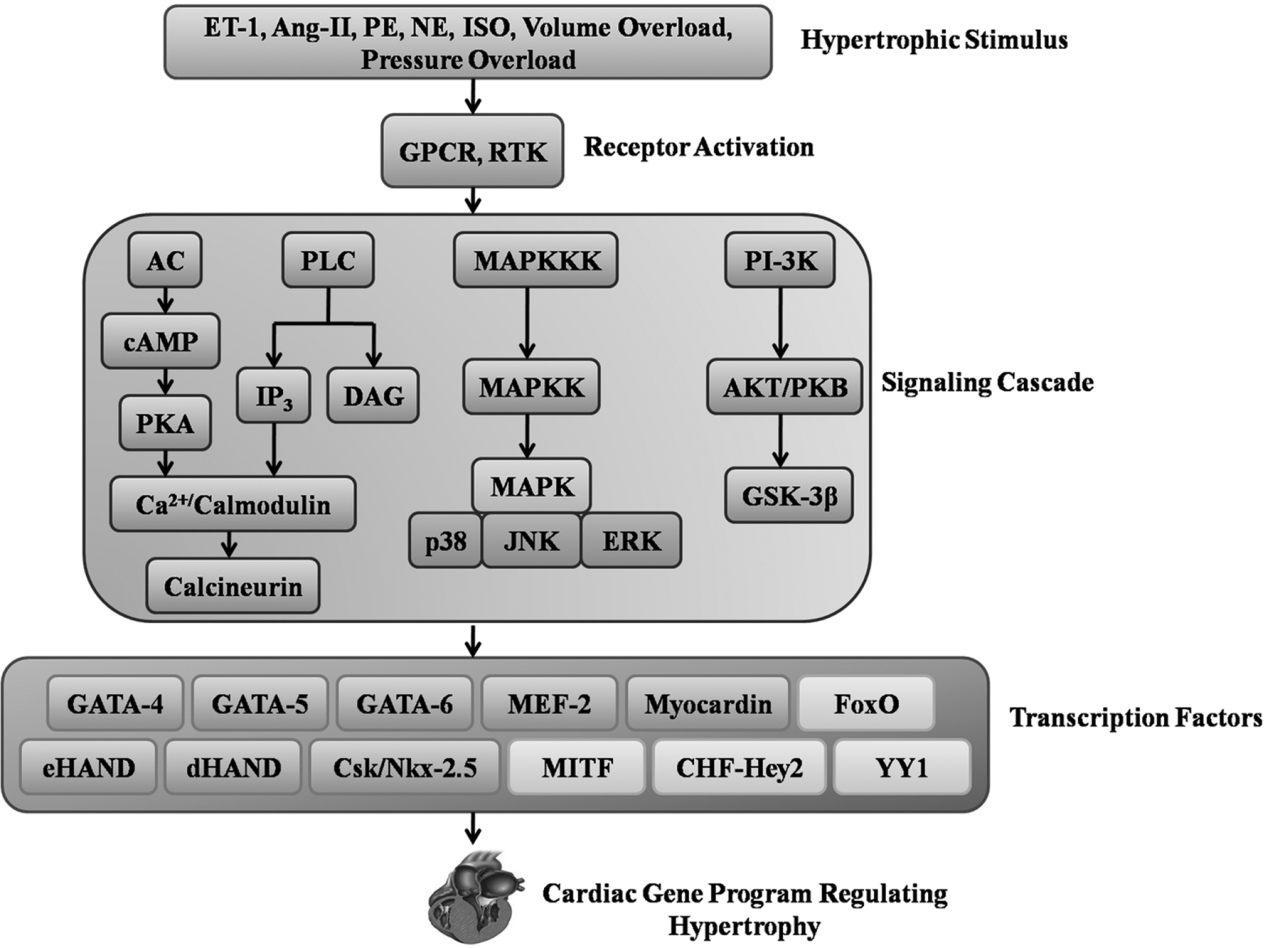
**Signaling pathways in cardiac hypertrophy leading to the transcriptional programming.** The hypertrophic stimulus activates its corresponding receptor leading to a complex signaling cascade. Ultimately different signaling cascades converge on to a common program targeting the activity of certain transcription factors. This leads to activation of cardiac gene program in the nucleus which manifests finally as the hypertrophic phenotype.

Cardiac hypertrophy is the cellular response to stress which helps terminally differentiated cardiac myocytes to sustain workload. Prolonged hypertrophy is maladaptive and associated with a significant thickening of ventricular walls and resulting in progression to heart failure [10]. It is characterized by an increase in cardiomyocyte size, enhanced protein synthesis, re-expression of fetal genes such as Atrial Natriuretic Peptide (ANP), B-type Natriuretic Peptide (BNP), β-Myosin Heavy Chain (β-MHC) etc. It also involves re-organization of sarcomeres and activation of transcriptional machinery (Fig. **1**) [11, 12]. Induction of hypertrophy is a result of the triggering of a complex signaling cascade which include Ca^2+^ - Calcineurin – NFAT signaling, G-Protein Coupled Receptor (GPCR) signaling, Mitogen Activating Protein Kinase (MAPK) signaling, PI3 Kinase signaling via receptor kinase etc. (Fig. **2**) [13]. 

Although numerous biomechanical forces and pathological conditions can result in myocardial dysfunction, it is hypothesized that there is a final common pathway in which transcriptional alterations occur in sarcomeric gene expression, and that this pathway is common to different forms of pathologic hypertrophy. This makes it possible to view cardiac hypertrophy as a gene regulatory disorder. There are numerous transcription factors which have been implicated to be involved in cardiac development and diseases (Table **1**). This review focuses on the transcription factors that are predominantly expressed in myocardium and regulate the expression of cardiac genes encoding proteins which further govern the structure and function of cardiomyocytes. These transcription factors represent promising targets for novel treatment strategies and therapeutic drugs which could act either by stimulating the transcription of specific genes required for a desired beneficial effect, or by inhibiting the transcription of genes affecting cardiac hypertrophy. Since this disease is regulated by multiple pathways and various transcription factors, which are also involved in cross talk with other signaling components, there is a need of thorough study in order to establish efficient therapeutic strategies.

## PRO-HYPERTROPHIC TRANSCRIPTION FACTORS

The transcriptional code that programs maladaptive cardiac hypertrophy includes a plethora of transcription factors which bind to the promoter of various cardiac muscle differentiation genes. Thus, an enhanced activity of these transcription factors is responsible for the hypertrophic response, and hence the name pro-hypertrophic transcription factors.

### GATA Transcription Factors Family

The GATA transcription factors are a family of double Zinc finger (CysX2-CysX17-CysX2-Cys) containing TFs and possess preferential binding to the specific consensus DNA binding sequence (A/T)GATA(A/G) [14]. The family consists of six members (GATA-1-6). GATA-1, 2, 3 regulate the hematopoietic stem cells [15-18]. GATA-4, 5, 6 are expressed in the heart and exercise regulation of the developmental processes including differentiation, migration of cardiomyoctes etc. [19]. They exhibit a high similarity in the amino acid sequence within the Zinc Fingers [20]. The C-terminal Zinc Finger is required for the DNA-binding activity, while the N-terminal finger interacts with Friend of GATA (FOG) and contributes to stability and specificity of DNA-binding.

GATA-4 is one of first transcription factors expressed in cardiac cells and plays an important role in transcriptional regulation during cardiac development and growth as well as in cardiac hypertrophy and heart failure [21, 22]. The GATA binding motif has been found within the regulatory regions of most cardiac expressed genes. GATA-4 induces several promoters that are activated during cardiac hypertrophy. β-MHC or Angiotensin II type I receptor (AT1) are induced in response to pressure overload [23]. Activation of BNP promoters due to Isoproterenol (ISO) or phenylephrine (PE) also require GATA binding site [24].

Initiation of a hypertrophic stimulus results in enhanced DNA-binding activity of GATA-4 which has been confirmed by several *in-vitro* and *in-vivo* studies [25-32]. The expression levels of GATA-4 are not altered by hypertrophic stimulation induced by pressure overload [26], Endothelin-1 (ET-1) stimulation [31] or α-adrenergic agonists [33]. Rather, mechanical stretch [25], ISO [29] and electrical stimulation [34] have been found to elevate GATA-4 mRNA levels in neonatal rat cardiac myocytes. As there is an increase in DNA-binding activity of GATA-4, it can be hypothesized that GATA-4 undergoes post–translational modifications affecting its interaction with other cofactors.

Hypertrophic stimulus is associated with activation of several signal transduction pathways which ultimately activate GATA-4. Protein phosphorylation is pivotal in regulation of cellular processes such as cell growth, division, differentiation and apoptosis. Similarly, it plays a vital role in regulation of cardiac hypertrophic response through GATA-4. Pressure overload, ISO, PE, ET-1, Angiotensin-II, Phorbol 12-Myristate 13-Acetate (PMA), each induced activation of GATA-4 by phosphorylation [26, 29-33, 35-37]. PE induced activation is coupled with serine phosphorylation of GATA-4. Extracellular signal-regulated Kinase-2 (ERK-2) directly phosphorylates ser-105 in GATA-4 in cultured cardiomyoctes and dominant negative GATA-4 attenuated activated MEK1-induced myocyte growth in culture suggesting an essential role of ERK signaling in regulating hypertrophic response through GATA-4 [36].

In addition, induction by PE leads to Rho A mediated sarcomeric reorganization via p38 MAPK and promotes direct phosphorylation of GATA-4 at ser-105 *in vivo* [38]. Intracellular intermediates also regulate GATA-4 activity. For example Protein Kinase C (PKC) and JAK-STAT pathways converge on GATA-4 where PKC phosphorylation enhances GATA-4 DNA-binding activity and STAT-1 functionality, and bring about physical interaction with GATA-4 to activate other promoters [39].

GATA-4 activity is negatively regulated by Glycogen Synthase Kinase -3β (GSK-3β) through nucleo-cytoplasmic shuttling of GATA-4. In its activated state, GSK-3β phosphorylates the N-terminal domain of GATA-4 protein. GATA-4 phosphorylation leads to a decrease in its nuclear levels through the nuclear exportin Crm1 [29]. Consequently, there is a fall in the GATA-4 assisted transcriptional activity due to its unavailability to bind to its cognate DNA sequence in the nucleus. Phosphatidyl Ionositol 3 Kinase (PI3-Kinase) activation inhibits GSK-3β, thereby preventing the nuclear export of GATA-4, and hence leading to the expression of genes of the hypertrophic pathway.

Activity of GATA-4 is a result of cooperation with a number of other transcription factors and co-activators. p300 possesses intrinsic histone acetyltransferase (HAT) activity which is required for synergistic transcriptional activation of GATA-4. The C-terminal Zinc-Finger domain of GATA-4 is known to interact with various transcriptional activators such as GATA-6, MEF-2, NFAT, Nkx-2.5, SRF, dHAND and YY1 [40-46], whereas the N-terminal domain of GATA-4 activity is regulated by several signaling pathways as a result of hypertrophic stimulation and is involved in development of cardiac hypertrophy. In recent studies, it was found that GATA-6 transcription factor is also necessary for regulating cardiac hypertrophic response. The transcriptional potency of GATA-6 is hypothesized to be different from GATA-4 [47].

The GATA transcription factors are well characterized and studied transcription factors involved in hypertrophic cardiomyopathy. Furthermore, they are involved in interaction with many other signaling components of various hypertrophic pathways. Therefore, targeting the transcription factors of this family and their interaction with other signaling components provides an attractive therapeutic opportunity in the treatment of cardiac hypertrophy.

### Myocyte enhancer factor-2 Family

The myocyte enhancer factor-2 (MEF-2) transcription factors are a family containing a MADS (MCM1 agamous, deficiens and SRF) domain and an adjacent MEF-2 specific domain which together bind to their cognate DNA sequence CAT(A/T)GTA(G/A) [48, 49]. The family consists of four members (MEF-2A, MEF-2B, MEF-2C and MEF-2D). MEF-2A and 2D are majorly involved in the regulation of immune system and striated muscles. MEF-2C is known to have an essential role in differentiation of myocardial cells and postnatal growth of myocardium [50].

The MEF-2 binding motif has been identified within promoter region of cardiac genes [51]. MEF-2 transcription factors are associated with the regulation of genes expressed during cardiac hypertrophy. Similar to GATA-4, the DNA binding activity of MEF-2 has been found to increase due to pressure and volume overload [52].

Hypertrophic signaling pathways that lead to the activation of MEF-2 transcription factors chiefly include phosphorylation by p38 MAPK [53], ERK5 also known as MAPK1 [54, 55] and PI3K-Akt pathways [56, 57]. Besides, MEF-2 is an important effector of Ca^2+^ signaling owing to activation of Ca^2+^ - binding proteins, calcium calmodulins (CaMs) and their downstream effector calmodulin kinases (CaMKs) and calcineurin which induce cardiac hypertrophy [44, 58]. CaMKs are known to phosphorylate class II HDACs (HDAC-4,5,7,9) and inactivate them which otherwise repress the action of MEF-2 [59]. Phosphorylation of HDACs by CaMKs results in recruitment of intracellular chaperones 14-3-3 to dissociate the HDAC-MEF-2 complex [60-62]. This leads to nuclear export of HDACs and further activation of MEF-2 by binding to co-activators with HAT activity such as CREB Binding Protein (CBP) and p300 [63-66]. Calcineurin exposes the nuclear localization signals on NFAT and results in its nuclear import. This further promotes the formation of a complex between MEF-2 and NFAT leading to a synergistic activation of the target genes resulting in hypertrophy [67]. The exact mechanism is still undetermined.

MEF-2 also interacts with various co-activators such as GATA-4 [43], NFAT [67], MyoD [68], Smad proteins [69]. This cross talk makes it an essential component of the hypertrophic pathway and an effector triggering the genes resulting in manifestation of hypertrophic phenotype. Thus, therapeutic strategies can be developed which are able to target such an effector and can circumvent the hypertrophic events.

### Csx/Nkx-2.5

Csx/Nkx-2.5 is a homeobox containing gene identified originally as a vertebrate homolog of the *Drosophila *gene* tinman* [70, 71]. A non-functional mutant gene exhibits complete loss of heart formation in *Drosophila*, which establishes its importance in heart development. Further, the expression of Csx/Nkx-2.5 is restricted to heart in various vertebrate species from Zebra fish to Humans [45]. The homeodomain of Csx/Nkx-2.5 has a helix-turn-helix motif that binds to the specific consensus DNA sequence T(C/T)AAGTG [72]. Pressure overload, PE, ISO and other hypertrophic stimulus upregulate expression of Csx/Nkx-2.5 along with upregulating the expression of its target genes. The transcriptional activity is modulated through physical interactions with transcription factors such as GATA-4, MEF-2, eHAND and other co-activators [40, 45, 73, 74]. A synergy between Csx/Nkx-2.5 and GATA-4 activated many cardiac gene promoters such as those of ANP, a well-established marker gene for cardiac hypertrophy. GATA motif is located near the Csx/Nkx-2.5 binding element NKE2 (Nkx-2.5 response element-2). Co-transfection of expression plasmids of Csx/Nkx-2.5 and GATA-4 synergestically activated ANP promoter contained in the reporter gene [45]. Overall, it can be implicated that Csx/Nkx-2.5 has enhanced activity under hypertrophic conditions which regulates the cardiac gene program in hypertrophied hearts. This makes them an attractive target for the development of therapeutic strategies.

### SRF and Myocardin

Serum Response Factor (SRF) is a MADS-box transcription factor binds to the CArG box DNA consensus CC(A/T)_6_GG [75]. These binding sites are present in the promoter region of cardiac genes involved in hypertrophy. An SRF transcriptional co-activator called Myocardin is a transcription factor that is expressed specifically in smooth and cardiac muscle cell lineages and transactivates the genes containing a CArG box [76, 77]. Myocardin belongs to SAP domain family and does not bind to DNA alone, rather it forms a stable ternary complex with SRF which is bound to DNA. Myocardin has been found to have critical role in heart development [76]. It also participates as a molecular switch in controlling genes associated to smooth muscle cell proliferation and differentiation [78]. The pro-myogenic activity of myocardin requires association with SRF and is augmented by homodimerization that provides a molecular basis for the cooperativity among CArG boxes required for smooth muscle gene activator [79]. Induction of hypertrophy triggers the transcriptional activity of myocardin. Overexpression of myocardin in neonatal rat cardiomyocytes induces hypertrophy. Myocardin-induced hypertrophy is accompanied by cellular enlargement, elevated ANF expression and organization of sarcomeres. It is hypothesized that myocardin activity is induced by hypertrophic signaling through a post-translational modification [80]. These evidences suggest that myocardin is a nuclear effector of cardiac signaling pathways that connect signals to a transcriptional program for cardiac remodelling involved in hypertrophy. Targeting this nuclear effector may break the link between the two and prevent the manifestation of the disease. However, development of therapeutic strategies targeting these factors need much more elaborate studies to be established. 

### HAND Transcription factors

These are basic helix-loop-helix transcription factors having role in cardiac development and diseases [81]. Mainly two transcription factors belong to this family. The gene HAND1 (synonymous to eHAND, Thing 1, Hxt**) **is selectively expressed in ventricles, predominantly in the right ventricle. The gene HAND2 (synonymous to dHAND, Thing 2, Hed**)** is expressed both in atria and ventricles [82]. The expression of HAND genes in hypertrophy is obscure. It has been found that expression levels are unchanged in right ventricle samples but both were significantly elevated in left ventricle samples [83, 84]. In a PE-induced hypertrophic mouse, downregulation of HAND1 in left ventricle and HAND2 in right ventricle was observed [85]. The candidate genes under the control of these transcription factors are not well studied but it is shown that these transcription factors act as co-activators along with other cardiac transcription factors to synergistically transactivate cardiac genes. HAND2 is essential for cardiac fusion pathway through negative modulation of Fn1 levels [86]. Also, on binding to p300, HAND2 interacts with GATA-4 to activate promoters of ANP, BNP, and α-MHC. HAND1 also activates ANP gene by interacting with Csx/Nkx-2.5 [87]. Since not much is known about this transcription factor, revelation of its role in a much more detailed manner is required before proceeding towards therapeutic applications targeting these factors.

Besides these, scientists are exploring more transcription factors which can be involved in cardiomyopathies. Of these, recently the TEA domain factors have found to be implicated in cardiac specific gene expression and the hypertrophic response of primary cardiomyocytes to hormonal and mechanical stimuli. All TEAD family members contain an evolutionarily conserved 72-amino acid DNA binding domain (TEAD) and bind to the sequence 5′-CATTCC(T/A)-3′ located in the promoter/enhancer region of numerous striated and smooth muscle genes. It also regulates the gene expression by combinatorial interactions with adjacently bound transcriptional regulators and co-activators [88]. Most studies have suggested its role in development and its role in hypertrophy is recently being investigated. Nuclear factor of activated T cells (NFAT) is another crucial transcription factor involved in hypertrophic pathways and majorly plays its role by working in coordination with other cardiac transcription factors. Activation of calcineurin by Ca^2+^ results in the dephosphorylation and nuclear translocation of cytoplasmic latent NFAT transcription factors [89]. It further induces the expression of hypertrophic genes, including brain natriuretic peptide (BNP) and interacts with other hypertrophic signaling cascades, supporting the concept that hypertrophy is controlled by crosstalking signaling networks [90]. They likely function as only a small component within a more comprehensive and integrated signaling network that ultimately directs the entire myocyte growth response.

## ANTI-HYPERTROPHIC TRANSCRIPTION FACTORS

The anti-hypertrophic effects in cardiomyocyes can be mediated via inhibitory pathways by transcriptional repressors or suppression of pro-hypertrophic pathways. The transcription factors that regulate these effects are anti-hypertrophic transcription factors. They help in preventing the occurrence of the hypertrophic state by promoting anti-hypertrophic events.

### FoxO 

The Forkhead/winged family of transcription factors is an essential factor involved in development. It is characterized by a conserved DNA binding domain known as Forkhead box (Fox) that targets a DNA binding sequence TTGTTTAC [91]. The Forkhead proteins are classified in 19 subfamilies (A-S) of which 3 members of FoxO – FoxO1, FoxO3 and FoxO4 have been found to be critical in maintaining cardiac function [92, 93]. 

FoxO transcription factors modulate response to stress conditions, cell-cycle progression, protein degradation and apoptosis [94, 95]. Post-translational modifications that include phosphorylation, acetylation, glycosylation and ubiquitination, along with association with co-activators such as Smad [96], Notch [97], β-Catenin [98] regulate the FoxO transcriptional activity which is generally associated with counteracting oxidative stress and promoting cell cycle arrest and apoptosis [99].

A number of signaling cascades are involved in cardiac hypertrophy of which the Ca^2+^-Calcineurin pathway has been found to be associated with FoxO transcription factors in regulating hypertrophic growth in cardiomyocytes [100]. Overexpression of either FoxO1 or FoxO3 leads to a decrease in calcium phosphatase activity that reduces agonist induced expression of calcineurin interacting protein [101, 102]. In addition to this, the nucleo-cytoplasmic shuttling mechanism of FoxO is controlled by PI3-Kinase-Akt signaling whereby PI3-Kinase phosphorylation causes Akt to translocate to the nucleus where it phosphorylates FoxO. This masks the nuclear localization sequence and promotes FoxO interaction with 14-3-3 proteins localized in the nucleus causing translocation of FoxO-14-3-3 complex to the cytoplasm where it is rendered inactive [103, 104]. Since FoxO is no longer present in the nucleus, it cannot mediate the transcription of its regulatory genes and is therefore not able to counteract the hypertrophic stress. Maintaining FoxO activity is thus critical in preventing Akt mediated cardiac hypertrophy. Understanding of its regulation will help in establishing its therapeutic role. These findings imply that therapeutic strategies may be established to augment FoxO nuclear relocation and transcriptional activity in order to circumvent the events leading to hypertrophy of the heart. 

### MITF

Microphthalmia Transcription Factor (MITF) is a basic helix-loop-helix leucine zipper DNA binding protein [105]. This transcription factor majorly regulates pigmentation of eyes and skin, and hearing in mice as well as human beings. Mutations in the gene result in deafness and poor pigmentation of eyes and skin in mice [106] and Waardenburg syndrome type II in human beings, characterized by hypopigmentation and deafness [107]. 

MITF is upregulated by phosphorylation through MAPK pathway [108-110]. β-Adrenergic agonists induce Protein kinase A (PKA) activity and PKA has been shown to induce MITF expression through cAMP response element binding protein (CREB) mediated as well as non CREB mediated mechanisms in melanocytes [111]. Its importance to MITF signaling in cardiomyocytes remains to be determined. It has been found that MITF is highly expressed in cardiac myocytes and its mutation leads to a diminished hypertrophic response to β-adrenergic stimulation [112]. The responsibility of MITF in the physiology of heart, its regulation and its participation in different signal transduction pathways in cardiac hypertrophy are the areas yet to be explored.

### YY1

YY1 is a 65-kDa multifunctional zinc finger ubiquitously expressed transcription factor, belonging to the human GLI-Kruppel family of nuclear proteins and essential for mammalian embryonic development [113, 114]. Identified as an initiator binding protein, it can either activate or inhibit transcription depending on the promoter context [115-118]. It is highly conserved across species and is involved in regulating cardiac disease and other cellular processes. YY1 differentially regulates a multitude of gene promoters by acting as repressor, activator, and/or initiator of transcription [119]. It has been found that YY1 competes with the action of SRF, suggesting the occupation of the former as a repressor of cardiac genes [120, 121]. Cellular environment, including co-activators and co-repressors, influence YY1 action. The CREB binding protein (CBP) belongs to a class of transcriptional cofactors that link upstream transactivators and the basal transcription machinery [122]. The interaction of Class II HDAC and HDAC5 with YY1 in muscle cells was found to be necessary for the repressor activity of YY1 in cardiac specific promoters [123]. Besides, overexpression of YY1 in cardiac myocytes under hypertrophic stimulus prevents nuclear export of HDAC5 and induction of fetal gene program [124,125]. These findings strongly suggest that YY1 functions as anti-hypertrophic factor and may be a protective mechanism against pathological hypertrophy. Since, it plays a crucial role by forming multi-component complexes with other co-activators/co-repressors, the gene expression can be controlled by targeting these complexes and this will serve as an additional target for therapy.

### CHF1/Hey2

CHF1 (also called Hey2, Hesr-2, Hrt2, HERP1 and *gridlock*) is a hairy related basic helix-loop-helix (bHLH) transcriptional repressor. It is known to be expressed during cardiovascular development and is involved in maintenance of ventricular function [126-128]. Its role in the progression of cardiomyopathies is uncertain. The transcription factor presumably functions through transcriptional repression of pro-hypertrophic genes. It has been hypothesized that since a loss in function of CHF1/Hey2 results in cardiomyopathy, conversely, a gain in its function will protect against the manifestation of cardiac hypertrophy [128]. Overexpression of CHF1/Hey2 in the myocardium was found to prevent phenylephrine-induced cardiac hypertrophy and expression of hypertrophy marker genes *in vivo* and *in vitro*. It was further established through reporter assays that CHF1/Hey2 can directly block activation of ANF, a hypertrophy-associated gene. Also, it was found to interact with GATA-4 in order to attenuate the hypertrophic transcriptional program [129]. Earlier studies suggest that interactions between hairy-related transcription factors and GATA-related transcription factors regulate important biological processes [130]. The relationship of CHF1/Hey2 to HDACs and other proteins associated with suppression of hypertrophy is yet to be determined. These findings may contribute to the anti-hypertrophic effects of CHF1/Hey2 and raise the possibility of directing the future therapies towards CHF1/Hey2 dependent pathways.

## CONCLUSION

Cardiac hypertrophy can thus be viewed as a gene regulatory disorder. Cardiac transcription factors are key players that regulate inducible gene expression in cardiac myocytes. The identification of transcription factors as the key regulatory molecules in this process and the analysis of their structure and function have revealed that these proteins are potential targets for therapeutic intervention. They organize the first crucial step in establishing gene function i.e. transcription of information in DNA into mRNA. Translation of mRNA results in synthesis of proteins that manifest hypertrophic conditions further leading to heart failure. Presently, the drugs with anti-hypertrophic activity target the outside-in signaling: i.e. they block hormones (catecholamines, angiotensin and aldosterone), calcium triggers (Ca^2+^ channel blockers) and target pathophysiological load (vasodilators and diuretics). Although this could be viewed as an indirect targeting of transcription factors but this approach lacks specificity and has limited potential given the redundancy and extensive cross-talk between upstream signaling cascades. Moreover, these therapies greatly vary in effectiveness depending on the signaling pathway targeted and in spite of such conventional therapy cardiomyopathies are an increasing cause of mortality worldwide. Developing drug molecules that will selectively affect particular transcription factors and suppress their activity can be of great therapeutic interest. Transcription factors have ligand-binding, dimerization, transeffector, DNA-binding, nuclear-localization, and regulatory domains, which may be targeted directly by small molecule drugs. Hence, the design of therapeutic agents targeted at pro-hypertrophic transcription factors regulating the initial expression should be the ultimate goal. So far, several agents have been shown to utilize the modulation and/or inhibition of NF-κB to carry out some part of their therapeutic purpose such as glucocorticoids, nonsteroidal anti-inflammatory agents (NSAID), vitamin E, curcumin, thiols, cyclosporin, rifampicin, dithiocarbamates, methotrexate, thalidomide, leflunomide and various fungal and bacterial metabolites. Another approach is promoting the activity of anti-hypertrophic transcription factors. This will provide a protective mechanism to the cells in order to prevent further hypertrophic derangement of gene expression. Other promising alternatives include artificial transcription factor mimics, which consist of normal transcription factor domains conjugated with synthetic compounds targeting DNA-binding domains or regulatory domains. Recent progress in molecular biology has provided new techniques to inhibit target gene expression. Antisense strategy that is complimentary to the mRNA of interest and therefore regulates transcription of disease related genes has important therapeutic potential. Novel molecular strategy in which synthetic double-stranded DNA with high affinity for a target transcription factor may be introduced into cells as “decoy” elements to bind the transcription factor and alter gene expression. Antisense oligodeoxynucleotides are useful tools for the anti-gene strategies for gene therapy and in the study of transcriptional regulation [131]. For instance, GATA decoy oligodeoxynucleotide treatment of cardiomyocytes blocked GATA-4 DNA binding activity in electrophoretic mobility shift analysis and decreased baseline expression of cardiac natriuretic peptides and GATA-dependent promoter activity [132]. For these approaches to be successful, novel delivery systems are necessary to be developed. Ongoing investigations will undoubtedly provide a clearer picture of the roles that transcription factors may play in molecular medicine. 

## Figures and Tables

**Table 1.** Transcription factors in cardiac hypertrophy

Transcription Factor	Type of domain	Binding sequence	Physiological Significance
Pro-Hypertrophic Transcription Factors			
GATA-Family	Double Zinc Finger	(A/T)GATA(A/G)	Hematopoesis and Cardiac Development
MEF-2	MADS domain	CAT(A/T)GTA(G/A)	Embryonic Development, differentiation and stress response
Csx-Nkx-2.5	Helix-turn-helix	T(C/T)AAGTG	Cardiogenesis
SRF & Myocardin	MADS-box	CC(A/T)6GG	Cell cycle regulation, Cardiac and smooth muscle gene expression
HAND	Helix-loop-helix	CANNTG	Cardiac and Vascular Development
TEAD	Helix-loop-Helix	CATTCC(T/A)	Fetal heart development and cardiac remodelling
NFAT	Rel homology region	(A/T)GGAAA(A/N)(A/T/C)N	Immune response, Cardiac and skeletal muscle development
Anti-Hypertrophic Transcription Factors			
FoxO	Forkhead box	TTGTTTAC	Cell growth, proliferation and differentiation
MITF	Helix-loop-helix Leucine Zipper	CACATG	Melanocyte and osteoclast development
YY1	Zinc Finger	CGCCATNTT	Histone modification for promoter regulation
CHF1/Hey2	Basic helix-loop-helix	CANNTG	Cardiac Development and ventricular function
